# Perceived blood glucose regulation after menopause: a cross-sectional survey in women with type 1 diabetes in the Netherlands

**DOI:** 10.1007/s00125-025-06518-z

**Published:** 2025-08-16

**Authors:** Esther M. Speksnijder, Suat Simsek, Peter H. Bisschop, Dirk Jan Stenvers, Sarah E. Siegelaar, MenoPause Consortium

**Affiliations:** 1 Department of Endocrinology and Metabolism, Amsterdam Gastroenterology Endocrinology Metabolism, Amsterdam Movement Sciences, Amsterdam UMC: location University of Amsterdam, Amsterdam, the Netherlands; 2 Department of Internal Medicine, Northwest Clinics, Alkmaar, the Netherlands; 3 Department of Endocrinology and Metabolism, Amsterdam Gastroenterology Endocrinology Metabolism, Amsterdam UMC: location Vrije Universiteit Amsterdam, Amsterdam, the Netherlands; 4 Department of Endocrinology and Metabolism, Amsterdam Gastroenterology Endocrinology Metabolism, Amsterdam UMC: location University of Amsterdam, Amsterdam, the Netherlands

**Keywords:** Carbohydrate metabolism, Clinical diabetes, Human, Insulin resistance, Insulin sensitivity, Other hormones/action

## Abstract

**Aims/hypothesis:**

Women with type 1 diabetes experience changes in insulin requirements in pregnancy and throughout the menstrual cycle. It remains to be explored whether women with type 1 diabetes perceive changes in glucose regulation during and after the menopausal transition, another period of marked hormonal change in a woman’s life.

**Methods:**

We conducted a cross-sectional survey to investigate whether women with type 1 diabetes perceive changes in glucose regulation after their final menstrual period. The online questionnaires were distributed through advertisements in hospitals and through online platforms for people living with type 1 diabetes in the Netherlands. Postmenopausal women (≥1 year of amenorrhoea) with type 1 diabetes, aged 45–65 years, were included. Participants with primary amenorrhoea, premenopausal hysterectomy or a postmenopausal diabetes diagnosis were excluded from the study. The primary outcome was the extent to which participants perceived changes in their glucose regulation following their final menstrual period, assessed using a five-point Likert scale. Menopausal symptom severity was estimated using the Greene climacteric scale (GCS).

**Results:**

Questionnaires from a total of 159 women were eligible for inclusion. Participants had a mean age of 54.9 years (SD 3.8), a mean diabetes duration of 30.3 years (SD 12.8), and had their final menstrual period at a mean age of 50.1 years (SD 5.0). Overall, 67.4% of participants reported moderate to severe postmenopausal changes in glucose regulation. Increased blood glucose levels were perceived by 41.9% of participants, 19.6% perceived lower glucose levels and 38.5% perceived no change in blood glucose levels. For fluctuations in glucose levels, 55.0% experienced more fluctuations and 18.1% experienced less fluctuation. More hyperglycaemic events were experienced by 61.6% of participants, while 38.5% experienced more hypoglycaemic events and 28.0% experienced fewer hypoglycaemic events. Reported menopausal symptoms were more severe after the final menstrual period compared with before the final menstrual period (mean GCS score ± SD: 18.8±9.9 vs 11.7±8.3, *p*<0.001). An increase in postmenopausal symptom severity score was associated with an increase in the odds of perceiving postmenopausal changes in glucose regulation, with an adjusted OR of 1.04 (95% CI 1.01, 1.08; *p*=0.014). A total of 57.2% of participants had a global Pittsburgh sleep quality index (PSQI) score >5, indicating poor sleep quality. Poor sleep quality was not associated with perceived glycaemic changes after menopause (global PSQI >5) (adjusted OR 1.10; 95% CI 0.58, 2.08; *p*=0.731).

**Conclusions/interpretation:**

Approximately two-thirds of women with type 1 diabetes perceive changes in their glucose regulation after menopause, including subjective changes in the number of hyperglycaemic and hypoglycaemic events. Changes in perceived glucose regulation were associated with the severity of reported menopausal symptoms. These results highlight the need for awareness among medical professionals treating women with type 1 diabetes, as those going through the menopausal transition may experience changes in glucose metabolism, which can affect their diabetes management.

**Data availability:**

The data that support the findings of this study are available on request from DataVerseNL with the identifier https://doi.org/10.34894/84QJOO.

**Graphical Abstract:**

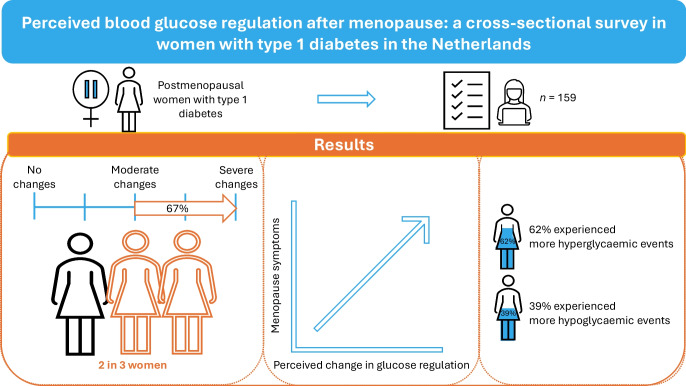

**Supplementary Information:**

The online version of this article (10.1007/s00125-025-06518-z) contains peer-reviewed but unedited supplementary material.


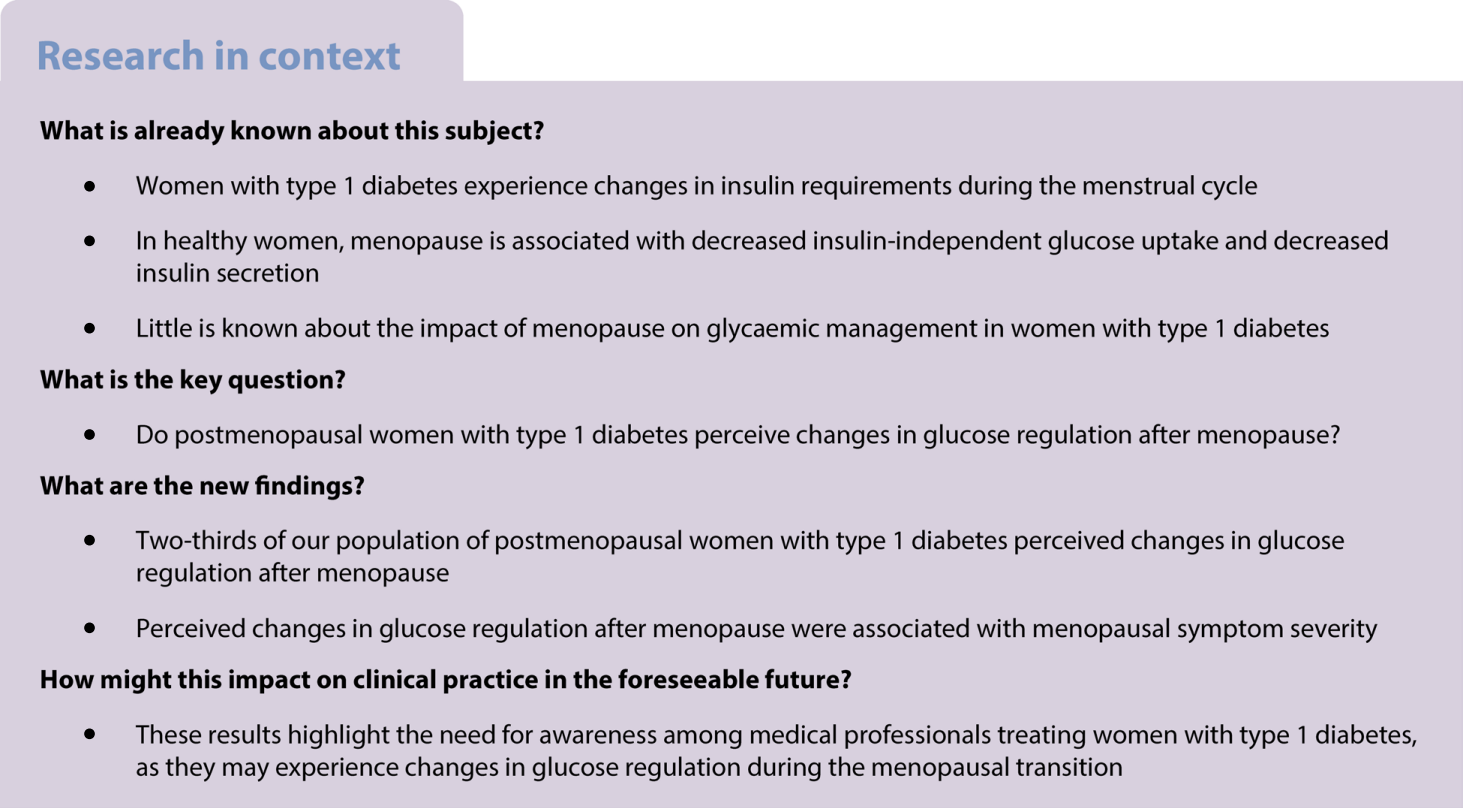


## Introduction

Type 1 diabetes is an autoimmune disease that affected approximately 8 million people worldwide in 2021 [1]. The management of type 1 diabetes requires frequent assessment and adjustment of insulin doses to meet the insulin requirements of the patient. Women with type 1 diabetes may experience changes in insulin requirement due to sex hormone fluctuations throughout life. It has been established that changes in glucose regulation occur within the menstrual cycle [2] and in pregnancy [3]. However, the impact of menopause on glycaemic management in women with type 1 diabetes remains to be explored.

Menopause occurs due to depletion of the ovarian oocytes, leading to a marked decrease in oestradiol and progesterone production, and the cessation of monthly menstrual cycles [4]. Animal studies have demonstrated that sex hormones, in particular oestradiol, affect glucose metabolism [2]. For example, ovariectomy in rodents rapidly decreases circulating oestradiol levels and induces insulin resistance [5]. In turn, oestradiol treatment restores insulin action and glucose tolerance in rodents [6]. In humans, women with type 1 diabetes perceive changes in glucose regulation across the menstrual cycle [7], reporting higher glucose levels during the luteal phase compared with the follicular phase. In addition, observations using continuous glucose monitoring (CGM) showed that premenopausal women with type 1 diabetes have a decreased time in range during the luteal phase compared with the follicular phase [2]. Given the rapid decrease in oestradiol and progesterone levels following menopause, we hypothesised that the menopausal transition may impact glucose regulation in early postmenopausal women with type 1 diabetes.

The decrease in oestradiol and progesterone concentrations around menopause leads to a variety of symptoms, including hot flushes, night sweats and sleep disturbances [8]. Sleep disturbances occur in 40–60% of perimenopausal and postmenopausal women [9]. Episodes of hot flushes and night sweats may interfere with sleep [9]. Moreover, the abundance of oestrogen receptors in the central brain clock (i.e. the area of the brain that orchestrates sleep–wake rhythms; the suprachiasmatic nucleus) suggests that a reduction in circulating oestrogens may affect circadian regulation of sleep [10]. Poor sleep quality has been associated with a negative impact on glycaemic management in people with type 1 diabetes [11]. Therefore, sleep disturbances around the menopausal transition may have important implications for women with type 1 diabetes, in addition to the effects of reduced circulating oestrogens alone.

To explore whether women with type 1 diabetes perceive changes in glucose metabolism after menopause, we performed a cross-sectional survey study in postmenopausal women with type 1 diabetes in the Netherlands. The primary aim of the survey was to determine to what extent postmenopausal women with type 1 diabetes mellitus perceive changes in glucose regulation after menopause. Furthermore, we aimed to identify the relationship between perceived changes in glucose regulation and menopausal symptoms. Finally, we assessed sleep quality in postmenopausal women with type 1 diabetes.

## Methods

### Study design

We developed a cross-sectional survey to explore whether women with type 1 diabetes perceive changes in glucose regulation after menopause. The survey was developed in the Dutch language by the study authors. To enhance survey quality, the questionnaire was tested using cognitive interviews in three women with type 1 diabetes [12]. Cognitive interviews rely upon a four-item model, introduced by Tourangeau et al [13]. In accordance with the four-item model, the three women were asked, for each new questionnaire item: (1) to explain the question in their own words; (2) what their first response would be to the question, and whether they felt they could adequately answer the question based on their memory; (3) whether they found the question difficult; and (4) whether they felt their first response answer was available among the answer options. Based on the feedback from the testing participants, the questionnaire was adapted after each cognitive interview. The demographic characteristics of the three testing participants are given in the electronic supplementary material (ESM) Table 1. The three participants involved in the testing of the survey were excluded from the study results.

The final version of the questionnaire contained six sections: (1) demographic characteristics (14 items); (2) menstruation and bleeding (5 items); (3) perceived changes in glucose regulation (13 items); (4) the Greene climacteric scale before and after menopause (2×21 items) and a question about hormone therapy use (1 item); (5) the ultra-short Munich chronotype questionnaire (7 items); and (6) the Pittsburgh Sleep Quality Index (15 items). The survey contained a total of 97 items and the estimated completion time was approximately 20 min. The questionnaire was completed in chronological order: each section needed to be fully completed before proceeding to the next section. The English translation of the survey is presented in ESM Appendix 1. We assessed the internal consistency reliability of the newly developed questionnaire items using Cronbach’s alpha calculations.

### Setting

Data were collected using convenience sampling. Questionnaires were distributed through advertisements (posters/flyers) in 41 hospitals in the Netherlands and on three online platforms for people living with type 1 diabetes in the Netherlands. The advertisements provided a QR code or link to access the online survey. The questionnaires were filled in anonymously and completed online in an electronic database (Castor EDC system [14]). Data were collected between 29 August 2022 and 1 October 2024. All participants provided digital informed consent before initiating the questionnaire. This study was carried out in accordance with the principles of the Declaration of Helsinki 2008. The institutional review board at the Amsterdam University Medical Centres (Amsterdam UMC: location AMC) reviewed the study protocol and determined that the study is not subject to the Dutch Medical Research Involving Human Subjects Act.

### Participants

Postmenopausal women (≥1 year of amenorrhoea) with type 1 diabetes, aged 45–65 years, were included in the study. The source population consisted of women with type 1 diabetes in the Netherlands who were either recruited via one of the three online platforms for people living with type 1 diabetes (listed in the Acknowledgements section) or notified about the study by their physician at one of the 41 participating hospitals in the Netherlands. Women taking postmenopausal hormone therapy (HT) were allowed to participate in the study. Postmenopausal status and type 1 diabetes diagnosis were assessed using signalling questions at the start of the questionnaire. Participants reported their age at the final menstrual period, age at diabetes diagnosis and whether they had undergone a hysterectomy or ovariectomy. Women who reported having less than 1 year of amenorrhoea, and women with primary amenorrhoea, premenopausal hysterectomy or a postmenopausal diabetes diagnosis were excluded from the study analyses. Duplicate questionnaires were identified using the built-in duplicate checker tool in SPSS version 28 (IBM SPSS Statistics). Data on educational level and place of birth were collected. Race and ethnicity data were not collected, as Dutch regulations state that such data may not be obtained through anonymous questionnaires, which fall outside the scope of the Dutch Medical Research Involving Human Subjects Act.

### Outcomes

The primary outcome was perceived change in glucose regulation, assessed using a five-point Likert scale. The participants could indicate to what extent they perceived changes in glucose regulation after their final menstrual period compared with before their final menstrual period according to the following categories: (1) no change; (2) little change; (3) moderate change; (4) large change; or (5) huge change. Next, participants indicated whether their glucose levels after their final menstrual period compared with before their final menstrual period were: (1) much lower; (2) lower; (3) similar; (4) higher; or (5) much higher. We estimated menopausal symptom severity using the Greene climacteric scale (GCS) [8]. Participants retrospectively reported the extent to which they experienced menopausal symptoms before and after their final menstrual period: not at all (score 0); a little (score 1); quite a bit (score 2); or extremely (score 3). The sum of the menopausal symptom scores estimates menopausal symptom severity, with a higher score indicating higher severity of menopausal symptoms (score range 0–63).

Secondary outcomes were the perceived change in fasting glucose levels, the perceived change in HbA_1c_ levels, and the perceived change in insulin dosage and time in range. Sleep quality and chronotype were assessed using the Pittsburgh sleep quality index (PSQI) [15] and the ultra-short Munich chronotype questionnaire [16], respectively. The PSQI consists of seven components, each scored from 0 (no difficulty) to 3 (severe difficulty). The sum of the seven component scores produces a global score (range 0–21). A global PSQI score >5 indicates poor sleep quality, with a sensitivity of 89.6% and specificity of 86.5% [15].

### Sample size calculation

We estimated the prevalence of women with type 1 diabetes aged 45 to 65 years in the Netherlands, as approximately 13,200 [17]. Based on Cochran’s sample size formula [18], with a confidence level of 95% and an estimated proportion of 0.5 indicating change in glycaemic management after menopause, we required a sample size of 374 female participants with type 1 diabetes with an error margin of 5% with a minimum detectable effect size (Cohen’s *h*) of 0.15.

### Statistical analysis

Statistical analyses were performed using SPSS version 28 (IBM SPSS Statistics). Proportions are expressed as percentages, and continuous data are presented as means (± SD). We determined the frequencies (%) of perceived changes in glucose regulation using a five-point Likert scale. To determine the association between perceived changes in glucose regulation (five-point Likert scale) and the menopausal symptom burden, we performed ordinal regression analyses, with the ordinal five-point Likert scale as the dependent variable and the menopausal symptom score as the independent variable. We adjusted the ordinal regression analysis for age at final menstrual period, diabetes duration, BMI and use of oestrogen-containing HT. Exploratory subgroup analyses were performed to stratify for time since menopause, and to assess whether participants using an insulin pump vs participants using insulin pen therapy answered differently regarding perceived changes in glucose regulation. In addition, subgroup analyses were performed to assess whether participants using postmenopausal HT answered differently compared with non-HT users regarding perceived changes in glucose regulation. Paired continuous data were analysed using paired *t* tests or Wilcoxon signed-rank tests, depending on the distribution. Categorical data were compared using χ^2^ tests. A two-sided* p* value <0.05 was considered statistically significant.

Missing values in outcome variables were handled using multiple imputation in SPSS version 28. Methodologists consider multiple imputation to be a state-of-the-art technique because it improves accuracy and statistical power relative to other missing data techniques [19]. Visual inspection of the missing data showed a pattern for missingness, suggesting that missingness depended on other observed variables. Therefore, missing data were assumed to be missing at random (MAR) and not missing completely at random (MCAR). Three auxiliary variables with no missing values (BMI, age at final menstrual period and diabetes duration) were added to the imputation model to improve it. Next, we created 20 imputed datasets to estimate pooled values, as the percentage of incomplete cases was approximately 20% [20, 21]. Variables included in the imputation model are listed in ESM Table 2. Incomplete outcome variables were imputed using fully conditional specification and predicted mean matching, with a maximum iteration number of 50 to ensure convergence. Convergence plots were generated for each imputed item to investigate whether the imputed values had the expected variation between the iterations. The convergence plots showed a random pattern of convergence and stability in imputed values. The distribution of the original vs imputed values was compared using histograms. To assess the robustness of the multiple imputation strategy, a complete-case analysis was conducted (ESM Tables 3–5).

## Results

### Demographic characteristics

**Fig. 1:**
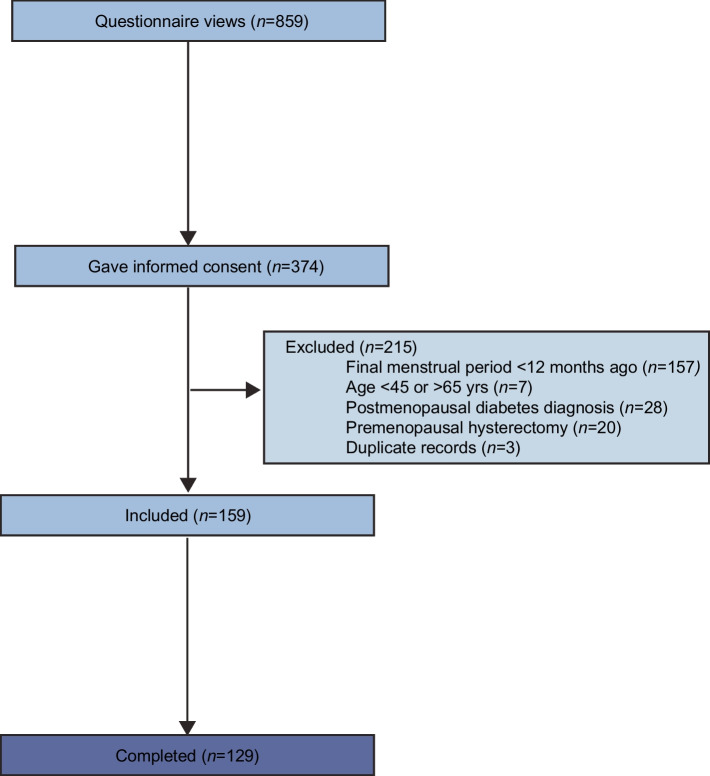
Flow diagram for survey inclusions

A total of 374 participants gave informed consent and initiated the questionnaire; 159 of these questionnaires were eligible for inclusion. The completion rate of the 159 eligible questionnaires was 81% (*n*=129) (Fig. 1). Because of the convenience sampling method used, the response rate could not be calculated. Missing values were handled using multiple imputation to yield a complete dataset for analysis of the outcome variables (*n*=159). ESM Table 2 shows the missing data rates for each imputed variable. As the actual sample size (*n*=159) was smaller than the intended sample size (*n*=374), we used Cochran’s sample size formula to calculate that the actual margin of error for the study results was 8%, rather than the pre-specified margin of error of 5% (18).

The 159 participants had a mean age of 54.9 years (SD 3.8), a mean diabetes duration of 30.3 years (SD 12.8), and had their final menstrual period at a mean age of 50.1 years (SD 5.0) (Table 1).

**Table 1:** Participant characteristics

Characteristic	Value (*N*=159)
Age, years	54.9±3.8
Age at final menstrual period, years	50.1±5.0
Diabetes duration, years	30.3±12.8
Ovariectomy	3 (1.9)
Educational level	
High school	16 (10.1)
College education	115 (72.3)
University	28 (17.6)
Place of birth	
The Netherlands	151 (95.0)
Belgium	2 (1.3)
Germany	3 (1.9)
Switzerland	1 (0.6)
Afghanistan	1 (0.6)
Missing	1 (0.6)
Method of insulin administration	
Pen	50 (31.4)
Pump	103 (64.8)
Pen and pump	6 (3.8)
Diabetes complications	54 (34.0)
Retinopathy	34 (21.4)
Foot complications	13 (8.2)
Neuropathy	15 (9.4)
Nephropathy	7 (4.4)
CVD	6 (3.8)
Other	15 (9.4)
Treating physician^a^	
General practitioner	2 (1.3)
Internal medicine specialist	156 (98.1)
Other	6 (3.8)
Subjective diabetes management	
Excellent	2 (1.3)
Very good	11 (6.9)
Good	90 (56.6)
Fair	46 (28.9)
Poor	10 (6.3)
BMI, kg/m^2^	26.2±4.5
HT use for menopausal symptoms^b^	22 (16.9)

Values are expressed as mean ± SD for continuous variables or *n* (%) for categorical variables

^a^*n*>159 as participants could provide multiple answers for this question

^b^*n*=130 due to 29 missing responses

### Perceived changes in glucose regulation

**Fig. 2:**
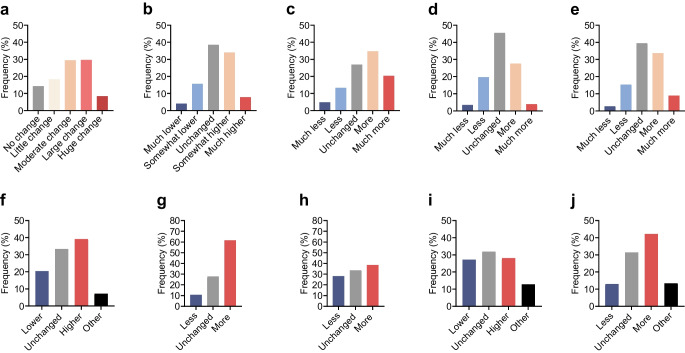
Perceived glucose regulation after the final menstrual period. The questionnaire items were: (**a**) ‘To which extent do you think your blood sugar levels have changed after your final menstrual period?’. (**b**) ‘Compared to before my final menstrual period, my blood sugars are…’; (**c**) ‘Compared to before my final menstrual period, my blood sugar levels fluctuate…’; (**d**) ‘Compared to before my final menstrual period, my insulin use is…’; (**e**) ‘Compared to before my final menstrual period, my insulin use fluctuates…’; (**f**) ‘Compared to before my final menstrual period, my HbA_1c_ is…’; (**g**) ‘Compared to before my final menstrual period I have… (hyperglycaemic events)’; (**h**) ‘Compared to before my final menstrual period I have… (hypoglycaemic events)’; (**i**) ‘Compared to before my final menstrual period, my time in range is…’; (**j**) ‘Compared to before my final menstrual period, my time in range fluctuates…’

Among the 159 participants, 67.4% observed moderate to severe postmenopausal changes in glucose regulation (Fig. 2a); 41.9% of participants reported increased blood glucose levels, 19.6% reported lower glucose levels and 38.5% reported no change in blood glucose levels (Fig. 2b).

A total of 55.0% of participants experienced more fluctuations in glucose levels and 18.1% experienced fewer fluctuations; 61.6% of participants experienced more hyperglycaemic events, 38.5% experienced more hypoglycaemic events and 28.0% experienced fewer hypoglycaemic events (Fig. 2c–h).

**Fig. 3:**
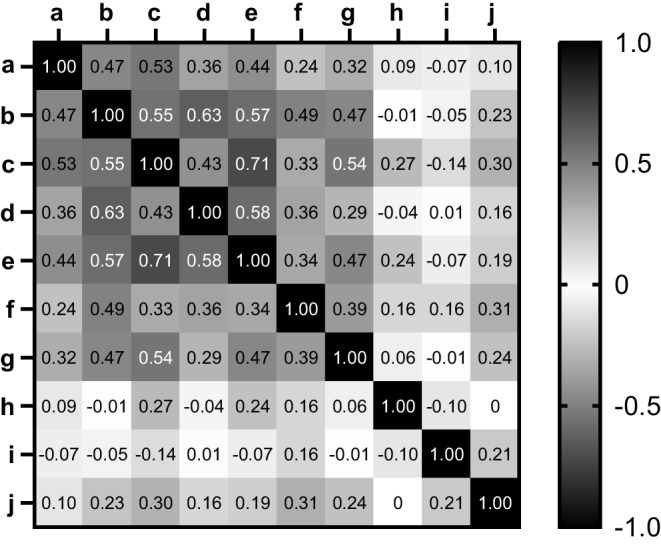
Spearman’s correlation coefficients for responses between individual questions. The corresponding questionnaire items are shown in Fig. 2

Cronbach’s alpha was 0.84 (95% CI 0.80, 0.88) for Fig. 2a–e; 0.63 (95% CI 0.51, 0.73) for Fig. 2f,i,j; and 0.64 (95% CI 0.49, 0.74) for Fig. 2g,h. Inter-item correlation coefficients are shown in Fig. 3.

**Fig. 4:**
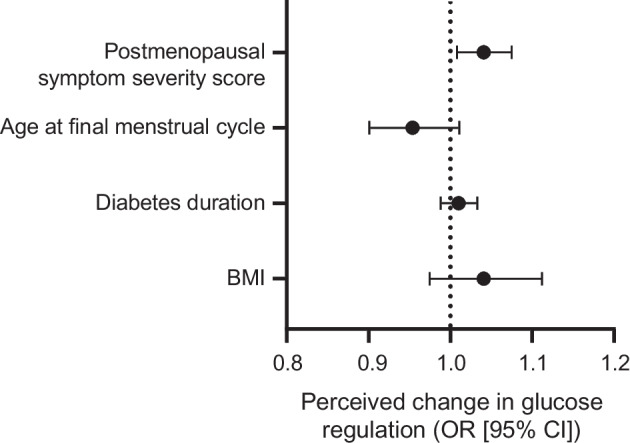
An increase in postmenopausal symptom severity score was associated with an increase in the odds of perceiving changes in glucose regulation (adjusted OR 1.04; 95% CI 1.01, 1.08; *p*=0.014) after adjustment for age at final menstrual cycle, diabetes duration, BMI and HT use. The adjusted odds ratios for the covariates age at final menstrual cycle (adjusted OR 0.96; 95% CI 0.90, 1.02; *p*=0.167), diabetes duration (adjusted OR 1.01; 95% CI 0.99, 1.04; *p*=0.365) and BMI (adjusted OR 1.05; 95% CI 0.98, 1.13; *p*=0.167) are also shown. The adjusted OR for HT use was 1.17 (95% CI 0.47, 2.87) but this is not shown due to the large width of the 95% CI, which hinders meaningful interpretation

Reported menopausal symptoms were more severe after the final menstrual period compared with before the final menstrual period (GCS scores 18.8±9.9 vs 11.7±8.3 [means ± SD], *p*<0.001). Pre- and postmenopausal symptom severity scores were comparable to those of a representative sample of the Dutch population [22]. An increase in GCS scores after the final menstrual period was associated with an increase in the odds of perceiving changes in glucose regulation (adjusted OR 1.04; 95% CI 1.01, 1.08; *p*=0.014; unadjusted OR 1.04; 95% CI 1.01, 1.07; *p*=0.015) (Fig. 4). An increase in GCS scores before the final menstrual period was also associated with an increase in the odds of perceiving changes in glucose regulation (adjusted OR 1.04; 95% CI 1.00, 1.08; *p*=0.044; unadjusted OR 1.05; 95% CI 1.01, 1.09; *p*=0.016). GCS sub-category scores are listed in ESM Table 4.

### Pittsburgh sleep quality index

The mean global PSQI score (± SD) was 5.99±2.42 (Table 2). Among the 159 participants, 57.2% had a global PSQI score >5, indicating poor sleep quality [15]. Participants with diabetes complications (34.0%) had a higher global PSQI score than participants without complications (mean difference 1.01; 95% CI 0.11, 1.92; *p*=0.03). Poor sleep quality was not associated with perceived glycaemic changes after menopause (global PSQI >5) (adjusted OR 1.10; 95% CI 0.58, 2.08; *p*=0.731; unadjusted OR 1.18; 95% CI 0.64, 2.17; *p*=0.600). The results from the ultra-short Munich chronotype questionnaire are given in ESM Table 6.

**Table 2:** Sleep quality characteristics for postmenopausal women with type 1 diabetes, assessed using the PSQI

PSQI item	Score (*N*=159)
Global PSQI score	5.99±2.42
C1: Subjective sleep quality	1.49±0.65
C2: Sleep latency	1.52±1.11
C3: Sleep duration	0.17±0.45
C4: Sleep efficiency	0.07±0.08
C5: Sleep disturbance	1.62±0.60
C6: Use of sleep medication	0.25±0.73
C7: Daytime dysfunction	0.90±0.73

Values are presented as mean ± SD. The score for each item ranges from 1 to 3

### Subgroup analyses

Participant responses regarding perceived changes in glucose regulation were not different between insulin pen users (*n*=50) and pump users (*n*=109), except for reported HbA_1c_ levels. A larger proportion of participants using an insulin pen reported a change in HbA_1c_ levels after menopause, in comparison with participants using an insulin pump (39.7% vs 19.8%, *p*=0.02).

We also grouped women based on time since their final menstrual period: ≤5 years (*n*=110) and >5 years (*n*=49). The proportion of women perceiving moderate to severe changes in glucose regulation was higher in the ≤5 years group vs the >5 years group (74.1% vs 52.7%, *p*=0.015). The proportion of participants reporting increased blood glucose levels was significantly higher in the ≤5 years group vs the >5 years group (51.8% vs 19.6%, *p*<0.001), and the proportion of participants experiencing more fluctuations in glucose levels was higher (61.4% vs 41.2%, *p*=0.047).

Use of HT during the menopausal transition for menopause-related symptoms was reported by 16.9% (*n*=22) of participants. Four participants had used both oral and transdermal HT, 14 participants had used only oral HT, and four participants had used only transdermal HT. Participants who used HT did not differ regarding perceived changes in glucose regulation compared with participants who did not use HT. However, postmenopausal symptom severity score was lower in the group using HT (mean difference −7.9; 95% CI −12.3, −3.4; *p*<0.001). Premenopausal symptom severity score was not significantly lower in the group using HT (mean difference −3.1; 95% CI −6.9, –0.6; *p*=0.101).

### Sensitivity analyses

We obtained comparable results when the analysis was restricted to complete cases only (ESM Tables 3–5). Excluding the three participants who underwent an ovariectomy also did not affect the study results.

## Discussion

This cross-sectional survey aimed to determine whether postmenopausal women with type 1 diabetes perceive changes in glucose regulation after menopause. We found that 67% of respondents reported experiencing moderate to severe changes in glucose regulation after their final menstrual period, including more hyperglycaemic and more hypoglycaemic events. Furthermore, higher menopausal symptom severity was associated with an increase in the odds of perceiving postmenopausal changes in glucose regulation. These results suggest that a substantial proportion of women with type 1 diabetes may perceive changes in glucose regulation after menopause, and that perceiving these postmenopausal changes in glucose regulation occurs more often in women with higher menopausal symptom severity.

Although 67% of participants experienced moderate to severe postmenopausal changes in glucose regulation, our results suggest that the experienced changes were not only attributed to experiencing higher glucose levels. Although 42% of participants reported experiencing increased glucose levels after menopause, changes also included decreased glucose levels, which were perceived in 20% of participants. In addition, 62% of participants experienced more hyperglycaemic events, while a lower number of participants reported higher overall glucose levels (42%) and higher HbA_1c_ (39%). A possible explanation for this discrepancy is that hyperglycaemic events are more closely related to glucose fluctuations than to overall glucose levels and HbA_1c_. In agreement with this explanation, there was a stronger correlation between experiencing glucose fluctuations and experiencing hyperglycaemic events (*r*=0.54) compared with the correlation between experiencing higher HbA_1c_ levels and experiencing hyperglycaemic events (*r*=0.39) (Fig. 3).

A recent prospective study from 2021 investigated postmenopausal changes in glucose metabolism in patients with type 1 diabetes using a large patient database. The authors identified 548 women with type 1 diabetes over the age of 55 years (postmenopausal) and 630 women with type 1 diabetes below the age of 45 years (premenopausal) [23] and found that postmenopausal women required a lower dose of basal insulin compared with premenopausal women with type 1 diabetes. However, information on age at the final menstrual period was not available and no CGM data were available to assess changes in glucose fluctuations. As our results suggest that women with type 1 diabetes may perceive changes in glucose fluctuations during the menopausal transition, further research is needed to objectively assess glucose fluctuations during the menopausal transition.

It is highly plausible that changes in oestradiol and progesterone concentrations influence glycaemic management in women with type 1 diabetes. Oestradiol and progesterone affect multiple metabolic tissues, influencing both systemic and local metabolism [24]. Animal studies have revealed beneficial effects of oestradiol on insulin sensitivity, while progesterone may induce insulin resistance [25–27]. The menopausal transition is associated with weight gain, increases in total body fat and visceral fat and decreased energy expenditure [5, 28], leading to adipose tissue expansion and chronic adipose tissue inflammation, increasing proinflammatory macrophage activity and increasing local concentrations of proinflammatory factors [29]. These changes further increase the risk for developing insulin resistance [25, 27, 29]. Although evidence regarding the influence of menopause on glycaemic management is limited, the influence of the menstrual cycle on glucose regulation in type 1 diabetes has been more extensively investigated. Women with type 1 diabetes observe changes in capillary glucose and insulin requirements across the menstrual cycle [7], and studies using CGM have identified higher nocturnal glucose concentrations, a lower number of postprandial glucose fluctuations and lower insulin requirements during the follicular phase compared with the luteal phase in women with type 1 diabetes [2, 30, 31]. These findings demonstrate that fluctuations of female sex hormone concentrations influence blood glucose regulation in people with type 1 diabetes. We hypothesise that fluctuations in oestradiol and progesterone concentrations during the menopausal transition necessitate more frequent adaptation of insulin doses, possibly due to changing insulin sensitivity. As fluctuations in sex hormones are unpredictable around the menopausal transition, women with type 1 diabetes may experience more difficulties in managing their glucose levels during the menopausal transition and early postmenopausal phase, compared with the premenopausal phase.

Our results demonstrated an association between the extent of perceiving changes in glucose regulation and menopausal symptom severity. As menopausal symptoms (e.g. hot flushes, night sweats, irritability and fatigue) can mimic symptoms of hypoglycaemia or hyperglycaemia [8], menopausal symptoms may be mistakenly attributed to fluctuations in blood glucose levels. The small proportion of participants who reported the use of HT (16.9%) did not differ regarding perceived changes in glucose regulation compared with participants who did not use HT. Nevertheless, reviews of the impact of oestrogen-containing HT in women with type 1 diabetes suggest that further investigation through randomised controlled trials is required [32, 33]. For metabolic safety, transdermal oestradiol may be preferred in women with type 1 diabetes, as it avoids the hepatic first-pass effect. The first-pass effect of oral oestrogens has been linked to increased plasma triglycerides/triacylglycerols, inflammation and coagulation factors [5].

Furthermore, menopausal symptoms may reduce sleep quality, which may negatively affect glycaemic management [11]. Although having poor sleep quality was not associated with perceiving glycaemic changes after menopause in our study, 57.2% of participants reported having poor subjective sleep quality. In addition, the presence of diabetes complications further aggravated sleep quality. A meta-analysis from 2024 based on 37 studies of 29,284 participants found a comparable prevalence rate of poor sleep quality (PSQI >5) in healthy postmenopausal women (50.8%; 95% CI 45.4, 56.3%) [34], while in an online questionnaire study in 267 Dutch adults with type 1 diabetes (female sex: 60% of participants; age 47±16 years [mean ± SD]), only 31% of participants fulfilled the criteria of having poor sleep quality (PSQI >5) [35]. Taken together, the findings suggest that postmenopausal women, with and without type 1 diabetes, experience worse sleep quality compared with the general adult population.

Compared with a recent cohort of people with type 1 diabetes in the Netherlands, the proportion of participants using an insulin pump and the median BMI values for the participants were similar to the mean values in our study [36]. However, participants from our study were older compared with other cohort studies, as we only included postmenopausal women in our study [36, 37]. This may also explain why the duration of diabetes was higher in our population compared with other cohorts [36, 37]. However, the percentage of participants with diabetes-related complications was slightly lower compared with other cohorts, suggesting that glycaemic management was more optimal in our population compared with the average population of type 1 diabetes patients. Furthermore, the use of an online survey may have affected the representativeness of the study population, as it requires a certain level of digital literacy. Additionally, women with stronger views or greater interest in the menopause and type 1 diabetes may have been more likely to participate.

A limitation of this cross-sectional survey was the use of convenience sampling. The convenience sampling approach is prone to response bias and reduced external validity. However, in this study, we aimed to explore the extent of postmenopausal changes in glucose regulation, and the convenience sampling results were sufficient to demonstrate the relevance of further investigating peri- and postmenopausal glycaemic changes in women with type 1 diabetes. Also, our sample size was smaller than the pre-specified sample size (*n*=374). We calculated that the true population proportions for our sample size (*n*=159) lie approximately 8 percentage points above or below our sample estimations. Our results demonstrated that approximately 67% of participants perceived changes in blood glucose levels after menopause. We argue that if 59–75% of women with type 1 diabetes perceive changes in glucose regulation after menopause, with a confidence level of 95%, these estimations remain clinically relevant. As we used multiple imputation, the margin of error for population estimates of the full study population (*n*=159) was ≤8%. However, as the number of participants was lower in the subgroup analyses, the margin of error for proportion estimates for these analyses was higher than 8% and these results should therefore be interpreted with caution.

To prevent the variability of question interpretation and enhance survey quality, we performed cognitive interviews with patient representatives. The Cronbach’s alpha values for the newly developed questionnaire were adequate for the questionnaire items in Fig. 2a–e,g; however, the questionnaire items in Fig. 2f,h–j could be further improved. Adding additional items that are conceptually similar to these questions in future studies may help improve internal consistency. Another limitation of the study was that the results are susceptible to recall bias. As we included participants aged 45–65 years, the questions are retrospective assessments about the time around the last menstrual period. These events could have occurred approximately 1–20 years prior to completing the survey. The proportion of women perceiving moderate to severe changes in glucose regulation was higher in women with a more recent menopause (≤5 years ago), supporting the potential presence of recall bias. To mitigate bias and to confirm whether menopausal perceived glycaemic changes may be confirmed with objective glycaemic measures, prospective cohort studies are needed to collect both subjective data (menopausal symptoms) and objective data (CGM) during the entire menopausal transition in women with type 1 diabetes. However, as the postmenopausal stage only becomes apparent 1 year after the final menstrual period, investigating menopausal glycaemic changes remains a challenge. Future prospective cohort studies investigating the impact of menopause on glycaemic management would require yearly follow-up visits, an adequate sample size (67% of women perceiving changes requires a sample size of approximately 332 participants), yearly questionnaires to assess menopause symptoms and subjective changes in glucose regulation, yearly HbA_1c_ measurements and annual extraction of CGM data for individual participants to be stored in a database to track changes in time in range.

### Conclusion

Approximately two-thirds of our study participants experienced changes in glucose regulation after menopause, comprising both more hyperglycaemia and more hypoglycaemia. These results highlight the need for awareness among medical professionals treating women with type 1 diabetes, as women undergoing the menopausal transition may experience changes in glucose metabolism that may impact their treatment objectives. Prospective studies are needed to assess whether these perceived changes are mirrored by laboratory-measured glycaemic outcomes.

## Supplementary Information

Below is the link to the electronic supplementary material.

ESM (PDF 2210 KB)

## Data Availability

The data that support the findings of this study are available on request from DataVerseNL with the identifier https://doi.org/10.34894/84QJOO.

## Abbreviations

CGM: Continuous glucose monitoring
GCS: Greene climacteric scale
HT: Hormone therapy
PSQI: Pittsburgh sleep quality index
