# Supplementary material for: Perceived blood glucose regulation after menopause: a cross-sectional survey in women with type 1 diabetes in the Netherlands
Source: Diabetologia. 2025 Aug 16;68(11):2499–510. doi: 10.1007/s00125-025-06518-z (PMC12534243; doi:10.1007/s00125-025-06518-z)
Supplement: Supplementary file 1 — ESM (PDF 2210 KB) [file 125_2025_6518_MOESM1_ESM.pdf]

ELECTRONIC SUPPLEMENTARY MATERIAL

SUPPLEMENTARY RESULTS

ESM table 1. Demographics of testing participants

**Participant no.**	**Age (years)**	**Age at final menstrual period (years)**	**Diabetes duration (years)**
1	44	Premenopausal	39
2	65	58	48
3	57	55	29

ESM table 2. Missing data rates of outcome variables included in multiple imputation. The percentage of missing values across the outcome variables varied between 0% and 22%. As participants completed the questionnaire in a fixed order, missing data was more frequent for questions in the later sections.

**Variable**	**Missing data rate in *N* (%)**
BMI	0 (0)
Age at final menstrual period	0 (0)
Diabetes duration	0 (0)
Perceived change in glucose regulation	6 (3.8)
Glucose levels	7 (4.4)
Glucose fluctuations	12 (7.5)
Insulin use	20 (12.6)
Insulin use fluctuations	20 (12.6)
HbA1c	9 (5.7)
Hyperglycaemia	19 (11.9)
Hypoglycaemia	17 (10.7)
Time in Range	11 (6.9)
Time in Range fluctuations	20 (12.6)
Greene Climacteric scale score premenopausal	24 (15.1)
Greene Climacteric scale score postmenopausal	29 (18.2)
Number of weekly working days	32 (20.1)
Sleep time (workdays)	34 (21.4)
Sleep time (free days)	32 (20.1)
Wake up time (workdays)	34 (21.4)
Wake up time (free days)	32 (20.1)
Daytime dysfunction score	33 (20.8)
Sleep disturbance score	32 (20.1)
Sleep duration	32 (20.1)
Sleep latency	35 (22.0)
Use of sleep medication	30 (18.9)
Subjective sleep quality	31 (19.5)

ESM table 3. Complete-case analysis of primary outcome variables. The results presented in the table below are based on participants who fully completed the listed questionnaire item. Proportions were calculated using the total number of completed cases for that item as the denominator. The results obtained using the imputed data are shown in Figure 2 in the manuscript.

**Questionnaire item (no. of cases)**	**Mean ± SD / *N* (%)**
Perceived change in glucose regulation (*n* = 153)	
No change	22 (14.4)
Little change	28 (18.3)
Moderate change	45 (29.4)
Large change	45 (29.4)
Huge change	13 (8.5)
Glucose levels (*n* = 152)	
Much lower	6 (3.9)
Somewhat lower	24 (15.8)
Unchanged	58 (38.2)
Somewhat higher	52 (34.2)
Much Higher	12 (7.9)
Glucose fluctuations (*n* = 147)	
Much less	7 (4.8)
Less	20 (13.6)
Unchanged	39 (26.5)
More	51 (34.7)
Much more	30 (20.4)
Insulin use (*n* = 139)	
Much less	5 (3.6)
Less	27 (19.4)
Unchanged	62 (44.6)
More	39 (28.1)
Much more	6 (4.3)
Insulin use fluctuations (*n* = 139)	
Much less	4 (2.9)
Less	21 (15.1)
Unchanged	54 (38.8)
More	47 (33.8)
Much more	13 (9.4)

HbA1c (*n* = 150)	
Lower	31 (20.7)
Unchanged	49 (32.7)
Higher	59 (39.3)
Other	11 (7.3)
Hyperglycaemia (*n* = 140)	
Less	15 (10.7)
Unchanged	39 (27.9)
More	86 (61.4)
Hypoglycaemia (*n* = 142)	
Less	40 (28.2)
Unchanged	48 (33.8)
More	54 (38.0)
Time in Range (*n* = 148)	
Lower	40 (27.0)
Unchanged	47 (31.8)
Higher	42 (28.4)
Other	19 (12.8)
Time in Range fluctuations (*n* = 139)	
Less	18 (12.9)
Unchanged	44 (31.7)
More	58 (41.7)
Other	19 (13.7)
Greene Climacteric Scale score	
Before final menstrual period (*n* = 135)	11.7 ± 8.3
After final menstrual period (*n* = 130)	18.8 ± 10.0

In the complete-case analysis, increasing perimenopausal and postmenopausal menopausal symptom severity scores were associated with an increase in the odds of perceiving changes in glucose regulation, with an adjusted odds ratio of 1.05 [95% CI 1.01 – 1.09], p = 0.019 and 1.04 [95% CI 1.01 – 1.08], p = 0.008, respectively.

Greene Climacteric Scale sub-scores

The Greene Climacteric Scale score[1] covers 5 domains: Psychological scale (range 0- 33), divided in two subscales: anxiety (range 0-18) and depression (range 0-15), somatic scale (range 0-21), vasomotor scale (range 0-6) and sexual dysfunction scale (range 0-3). The items and their respective scores are listed below in supplementary table 4.

ESM table 4. Greene Climacteric Scale subscores

**Item, range (*n*)**	**Before menopause, median score (IQR)**	**After menopause, median score (IQR)**	***p*-value**
Psychological, range 0-33 (*n*=133)	6 (3-10)	10 (5-15)	<0.001
Anxiety, range 0-18 (*n*=133)	3 (2-5)	6 (3-8)	<0.001
Depression, range 0-15 (*n*=133)	3 (1-5)	5 (2-7)	<0.001
Somatic, range 0-21 (*n*=130)	3 (1-5)	4.5 (2-7)	<0.001
Vasomotor, range 0-6 (*n*=130)	1 (0-2)	2 (1-4)	<0.001
Sexual dysfunction, range 0-3 (*n*=130)	0 (0-1)	1 (0.75-2)	<0.001

ESM table 5. Complete-case analysis of sleep quality characteristics of postmenopausal women with type 1 diabetes, assessed using the Pittsburgh Sleep Quality Index (PSQI)[2]. The results obtained using the imputed data are shown in Table 2 of the manuscript.

**PSQI Item**	***N***	**Mean (SD)**
Global PSQI score, mean (SD)	126	6.0 (2.44)
C1: Subjective sleep quality (range 0-3), mean (SD)	128	1.49 (0.65)
C2: Sleep latency (range 0-3), mean (SD)	129	1.52 (1.11)
C3: Sleep duration (range 0-3), mean (SD)	127	0.17 (0.46)
C4: Sleep efficiency (range 0-3), mean (SD)	127	0.08 (0.09)
C5: Sleep disturbance (range 0-3), mean (SD)	127	1.62 (0.60)
C6: Use of sleep medication (range 0-3), mean (SD)	129	0.26 (0.73)
C7: Daytime dysfunction (range 0-3), mean (SD)	126	0.90 (0.74)

Ultra-short Munich Chronotype Questionnaire (MCTQ)

The average sleep duration of participants was 08:00 h ± SD 00:57 min. Sixteen participants (12.6%) reported an average sleep duration of less than 7 hours. The average midpoint of sleep on free days corrected for sleep-debt accumulated during the workweek (MSFsc)[3] was 03:25 h ± SD 00:44 min (Supplementary table 6).

ESM table 6. Complete-case analysis of sleep characteristics of postmenopausal women with type 1 diabetes, assessed using the ultra-short Munich Chronotype Questionnaire.

**Item**	**N**	**Values**
Number of weekly working days, median (IQR)	127	4 (2-4)
Sleep time, mean (hh:mm) (SD)	127	23:25 (00:49)
Workdays	125	23:16 (00:51)
Free days	127	23:32 (00:56)
Wake up time, mean (hh:mm) (SD)	127	07:24 (00:56)
Workdays	125	06:56 (00:55)
Free days	127	07:56 (01:02)
Sleep duration, mean (hh:mm) (SD)	127	08:00 (00:57)
Workdays	125	07:41 (01:02)
Free days	127	08:24 (01:07)
Midpoint of sleep corrected for sleep debt on working days, mean (hh:mm) (SD)	127	03:25 (00:44)
Workdays	125	03:06 (00:43)
Free days	127	03:44 (00:48)

References

1. Greene JG. Constructing a standard climacteric scale. *Maturitas*. May 20 1998;29(1):25-31. doi:10.1016/s0378-5122(98)00025-5
2. Buysse DJ, Reynolds CF, 3rd, Monk TH, Berman SR, Kupfer DJ. The Pittsburgh Sleep Quality Index: a new instrument for psychiatric practice and research. *Psychiatry Res*. May 1989;28(2):193-213. doi:10.1016/0165-1781(89)90047-4
3. Roenneberg T, Kuehnle T, Juda M, et al. Epidemiology of the human circadian clock. *Sleep Med Rev*. Dec 2007;11(6):429-38. doi:10.1016/j.smrv.2007.07.005

ESM Appendix 1. Perceived blood glucose regulation after menopause: Survey*

*****The Greene Climacteric Scale (GCS) and Pittsburgh Sleep Quality Index (PSQI) questionnaires have been redacted for copyright reasons.

Survey 'Blood sugar regulation during menopause'

Blood sugar regulation during menopause - Introduction

Dear participant,

Menopause is a natural period in the life of a female, and is defined as the last menstrual period. Changes in the female sex hormones estrogen and progesterone occur around the menopause. These changes may also affect blood sugars. Little research has been done on this subject in women with type 1 diabetes. Researchers at the Amsterdam UMC are therefore investigating how women with type 1 diabetes experienced their blood sugar regulation around the menopause.

The Questionnaire

The study consists of a questionnaire. It takes about 20 minutes to complete the questionnaire. Participation is voluntary and anonymous. You can decide to stop your participation at any time. If you stop the questionnaire, the researchers are allowed to use the results that you have filled in up to that point. Because the data are anonymous, we are not able to trace back and delete your data after you have closed the questionnaire.

Do you have any questions about the study? In that case, send an e-mail to the doctor-investigator who is coordinating the study: Esther Speksnijder, e.m.speksnijder@amsterdamumc.nl.

On the next page you can indicate whether you agree to the terms of the study. Afterwards, the questionnaire will appear. Thank you very much for completing the questionnaire!

Kind regards,

Dr. Sarah E. Siegelaar, Dr. Dirk Jan Stenvers, Prof. dr. Peter H.L.T. Bisschop, Dr. Suat Simsek, and Esther M. Speksnijder

Blood sugar regulation during menopause - Informed consent form

Number	Question	Answers
	Consent form for participation in medical research	
1.1	Informed consent<br>*Field type*: Summary<br>*Variable name*: engels_toestemming<br>*Field required*: Not required	
1.2	Do you agree with the above statements?<br>*Field type*: Radiobutton<br>*Variable name*: engelsakkoord<br>*Field required*: Required<br>*Option group name*: englishYes/No	○ Yes<br>○ No

Blood sugar regulation during menopause - General questions

Number	Question	Answers
2.1	***If 'Do you agree with the above statements?' is equal to 'Yes' answer this question:***<br>Are you currently going through menopause or have you been through menopause?<br>*Field type*: Radiobutton<br>*Variable name*: engelsovergang<br>*Field required*: Required<br>*Option group name*: engelsYes/No/Dont know	◯ Yes<br>◯ No<br>◯ I don't know
2.1.1	***If 'Are you currently going through menopause or have you been through menopause?' is equal to 'I don't know' answer this question:***<br>I don't know, because:<br>*Field type*: Textfield<br>*Variable name*: engelsovergang_anders<br>*Field required*: Required	[ ]
	General questions	
2.2	***If 'Do you agree with the above statements?' is equal to 'Yes' answer this question:***<br>Which type of diabetes do you have?<br>*Field type*: Radiobutton<br>*Variable name*: engelstype_diabetes<br>*Field required*: Required<br>*Option group name*: engelstypediabetes	◯ Type 1 diabetes<br>◯ Type 2 diabetes<br>◯ Other
2.3	***If 'Do you agree with the above statements?' is equal to 'Yes' answer this question:***<br>What is your age? (in years)<br>*Field type*: Numeric field<br>*Variable name*: engelsleeftijd<br>*Field required*: Required<br>*Field min*: 16<br>*Field max*: 110<br>*Measurement Unit*: years	[ ] years
2.4	***If 'Do you agree with the above statements?' is equal to 'Yes' answer this question:***<br>What is your height (cm)<br>For example: 170 cm<br><br>*Field type*: Numeric field<br>*Variable name*: engelslengte<br>*Field required*: Required<br>*Field min*: 50<br>*Field max*: 230<br>*Measurement Unit*: cm	[ ] cm

2.5	***If 'Do you agree with the above statements?' is equal to 'Yes' answer this question:***<br>What is your weight? (kg)<br>*Field type*: Numeric field<br>*Variable name*: engelsgewicht<br>*Field required*: Required<br>*Field min*: 20<br>*Field max*: 500<br>*Measurement Unit*: kg	[ ] kg
2.6	***If 'Do you agree with the above statements?' is equal to 'Yes' answer this question:***<br>engelslengte_m<br>*Field type*: Calculation<br>*Variable name*: engelslengte_m<br>*Field required*: Not required	
2.7	***If 'Do you agree with the above statements?' is equal to 'Yes' answer this question:***<br>BMI<br>*Field type*: Calculation<br>*Variable name*: engelsBMI<br>*Field required*: Not required	
2.8	***If 'Do you agree with the above statements?' is equal to 'Yes' answer this question:***<br>What is your highest education level?<br>*Field type*: Dropdown<br>*Variable name*: engelsopleidingsniveau<br>*Field required*: Required<br>*Option group name*: engelsopleiding	○ No education<br>○ Primary school<br>○ Middle school/high school<br>○ Middle-level vocational education<br>○ High-level vocational education<br>○ University bachelor's degree<br>○ University master's degree<br>○ Doctoral degree
2.9	***If 'Do you agree with the above statements?' is equal to 'Yes' answer this question:***<br>What is your country of birth?<br>*Field type*: Textfield<br>*Variable name*: engelsgeboorteland<br>*Field required*: Required	[ ]
2.10	***If 'Do you agree with the above statements?' is equal to 'Yes' answer this question:***<br>How old were you when you were diagnosed with diabetes? (in years)<br>*Field type*: Numeric field<br>*Variable name*: engelsleeftijd_diagnose<br>*Field required*: Required<br>*Field min*: 0<br>*Field max*: 100<br>*Measurement Unit*: years	[ ] years

2.11	***If 'Do you agree with the above statements?' is equal to 'Yes' answer this question:***<br>How is your diabetes being treated at the moment? (Choose one or more answers)<br>Choose one or more answers<br><br>*Field type*: Checkbox<br>*Variable name*: engelsbehandeling_diabetes<br>*Field required*: Required<br>*Option group name*: engelsbehandeling	☐ With insulin<br>☐ With a diet<br>☐ With pills<br>☐ With other injections (such as GLP-1 agonists)
2.11.1	***If 'How is your diabetes being treated at the moment? (Choose one or more answers)' is equal to 'With insulin' answer this question:***<br>What do you use to administer insulin to yourself?<br>*Field type*: Radiobutton<br>*Variable name*: engels_spuit_pomp<br>*Field required*: Required<br>*Option group name*: injection/pump	◯ Insulin injections<br>◯ Insulin pump<br>◯ Both injections and an insulin pump<br>◯ Other
2.11.1.1	***If 'What do you use to administer insulin to yourself?' is equal to 'Other' answer this question:***<br>If other, please specify:<br>*Field type*: Textfield<br>*Variable name*: engelsdiabetes_complicaties_1<br>*Field required*: Required	
2.11.2	***If 'How is your diabetes being treated at the moment? (Choose one or more answers)' is equal to 'With pills' answer this question:***<br>Which pills do you take for your diabetes treatment?<br>*Field type*: Checkbox<br>*Variable name*: pills<br>*Field required*: Required<br>*Option group name*: Pills	☐ Metformin<br>☐ Sulfonylureas (for example: gliclazide, tolbutamide, glibenclamide, glimepiride)<br>☐ SGLT-2 inhibitors (for example: dapagliflozine)<br>☐ DPP-4 inhibitors (for example: sitagliptine)<br>☐ Semaglutide<br>☐ I don't know<br>☐ Other
2.11.2.1	***If 'Which pills do you take for your diabetes treatment?' is equal to 'Other' answer this question:***<br>If other, please specify:<br>*Field type*: Textfield<br>*Variable name*: pills_other<br>*Field required*: Required	
2.12	***If 'Do you agree with the above statements?' is equal to 'Yes' answer this question:***<br>Do you have any diabetic complications?<br>*Field type*: Radiobutton<br>*Variable name*: engelsComplicaties_ja_nee<br>*Field required*: Required<br>*Option group name*: englishYes/No	◯ Yes<br>◯ No

2.12.1	***If 'Do you have any diabetic complications?' is equal to 'Yes' answer this question:***<br>Which complications? (Choose one or more answers)<br>*Field type*: Checkbox<br>*Variable name*: engelsdiabetescomplicaties<br>*Field required*: Required<br>*Option group name*: engelscomplications	☐ Foot damage<br>☐ Eye damage<br>☐ Kidney damage<br>☐ Nerve damage<br>☐ Cardiovascular disease<br>☐ Other
2.12.1.1	***If 'Which complications? (Choose one or more answers)' is equal to 'Other' answer this question:***<br>If other, please specify:<br>*Field type*: Textfield<br>*Variable name*: engelsdiabetes_complicaties<br>*Field required*: Required	
2.13	***If 'Do you agree with the above statements?' is equal to 'Yes' answer this question:***<br>Who treats you for your diabetes? (Choose one or more answers)<br>*Field type*: Checkbox<br>*Variable name*: engelsbehandelaar<br>*Field required*: Required<br>*Option group name*: engelsbehandelaar	☐ General practitioner / Diabetes specialist nurse<br>☐ Internal medicine specialist (endocrinologist)<br>☐ Other
2.13.1	***If 'Who treats you for your diabetes? (Choose one or more answers)' is equal to 'Other' answer this question:***<br>If other, please specify:<br>*Field type*: Textfield<br>*Variable name*: engelsandere_behandelaar<br>*Field required*: Required	
2.14	***If 'Do you agree with the above statements?' is equal to 'Yes' answer this question:***<br>In general, would you say your blood sugars are:<br>*Field type*: Radiobutton<br>*Variable name*: engelsdiabetes_instelling<br>*Field required*: Required<br>*Option group name*: engelsExcellent-Poor	○ Excellent<br>○ Very good<br>○ Good<br>○ Fair<br>○ Poor

Blood sugar regulation during menopause - Questions about periods and bleeding

Number	Question	Answers
3.1	***If 'Do you agree with the above statements?' is equal to 'Yes' answer this question:***<br>Questions about periods and bleeding<br>*Field type*: Summary<br>*Variable name*: engels_1<br>*Field required*: Not required	
3.2	***If 'Do you agree with the above statements?' is equal to 'Yes' answer this question:***<br>Which statement about periods and bleeding is most true for you?<br>*Field type*: Radiobutton<br>*Variable name*: engelsmenstruation<br>*Field required*: Required<br>*Option group name*: periods	○ My final menstrual period was more than 12 months ago<br>○ My final menstrual period was less than 12 months ago or exactly 12 months ago<br>○ I have regular menstrual periods<br>○ I take contraceptives / I have an IUD<br>○ I take hormone replacement therapy<br>○ Other
3.2.1	***If 'Which statement about periods and bleeding is most true for you?' is equal to 'Other' answer this question:***<br>If other, please specify:<br>*Field type*: Textfield<br>*Variable name*: engelsanders_menstruation<br>*Field required*: Required	[ ]
3.3	***If 'Do you agree with the above statements?' is equal to 'Yes' answer this question:***<br>How old were you when you had your last menstrual period? (in years)<br>*Field type*: Numeric field<br>*Variable name*: engelsleeftijd_laatstemenstruatie<br>*Field required*: Required<br>*Field min*: 0<br>*Field max*: 100<br>*Measurement Unit*: years	[ ] years
3.4	***If 'Do you agree with the above statements?' is equal to 'Yes' answer this question:***<br>Have you had a surgical procedure where your uterus was removed?<br>*Field type*: Radiobutton<br>*Variable name*: engelsovariectomie_1<br>*Field required*: Required<br>*Option group name*: engelsYes/No/Dont know	○ Yes<br>○ No<br>○ I don't know

3.4.1	***If 'Have you had a surgical procedure where your uterus was removed?' is equal to 'Yes' answer this question:***<br>How old were you when your uterus was removed? (in years)<br>*Field type*: Numeric field<br>*Variable name*: engels_leeftijdovariectomie_1<br>*Field required*: Required<br>*Field min*: 0<br>*Field max*: 120<br>*Measurement Unit*: years	[ ] years
3.4.2	***If 'Have you had a surgical procedure where your uterus was removed?' is equal to 'I don't know' answer this question:***<br>I don't know. Please specify why:<br>*Field type*: Textfield<br>*Variable name*: engelsovariectomie_weetniet_1<br>*Field required*: Not required	[ ]
3.5	***If 'Do you agree with the above statements?' is equal to 'Yes' answer this question:***<br>Have you had one or more surgical procedures where both of your ovaries were removed?<br>*Field type*: Radiobutton<br>*Variable name*: engelsovariectomie<br>*Field required*: Required<br>*Option group name*: engelsYes/No/Dont know	○ Yes<br>○ No<br>○ I don't know
3.5.1	***If 'Have you had one or more surgical procedures where both of your ovaries were removed?' is equal to 'Yes' answer this question:***<br>How old were you when both ovaries were removed? (in years)<br>*Field type*: Numeric field<br>*Variable name*: engels_leeftijdovariectomie<br>*Field required*: Required<br>*Measurement Unit*: years	[ ] years
3.5.2	***If 'Have you had one or more surgical procedures where both of your ovaries were removed?' is equal to 'I don't know' answer this question:***<br>I don't know. Please specify why:<br>*Field type*: Textfield<br>*Variable name*: engelsovariectomie_weetniet<br>*Field required*: Not required	[ ]

Blood sugar regulation during menopause - Questions about blood sugar regulation

Number	Question	Answers
4.1	***If 'Do you agree with the above statements?' is equal to 'Yes' answer this question:***<br>Questions about your blood sugar levels<br>*Field type*: Summary<br>*Variable name*: engels_2<br>*Field required*: Not required	
4.2	***If 'Do you agree with the above statements?' is equal to 'Yes' answer this question:***<br>To which extent do you think your blood sugar levels have changed after your final menstrual period? (Possible answers: 1. No change, 2. Some change, 3. Moderate change, 4. Big change, 5. Huge change)<br>1. No change<br>2. Some change<br>3. Moderate change<br>4. Big change<br>5. Huge change<br><br>*Field type*: Slider<br>*Variable name*: engelsverandering_glucoseregulatie<br>*Field required*: Required<br>*Field min*: 1<br>*Field min Label*: No change<br>*Field max*: 5<br>*Field max Label*: A huge change	No change (1) ______ A huge change (5)
4.3	***If 'Do you agree with the above statements?' is equal to 'Yes' answer this question:***<br>Compared to before my final menstrual period, my blood sugars are: (Possible answers: 1. Much lower, 2. Somewhat lower, 3. No difference, 4. Somewhat higher, 5. Much higher) 1: Much lower 2: Somewhat lower 3. No difference 4. Somewhat higher 5. Much higher<br><br>*Field type*: Slider<br>*Variable name*: engelsglucose_hoger<br>*Field required*: Required<br>*Field min*: 1<br>*Field min Label*: Much lower after my final menstrual period<br>*Field max*: 5<br>*Field max Label*: Much higher after my final menstrual period	Much lower after my final menstrual period (1) ______ Much higher after my final menstrual period (5)

4.4	***If 'Do you agree with the above statements?' is equal to 'Yes' answer this question:***<br>Compared to before my final menstrual period, my HbA1c:<br>*Field type*: Radiobutton<br>*Variable name*: engelsHbA1c<br>*Field required*: Required<br>*Option group name*: higher/lower/the same	○ Did not change after my final menstrual period<br>○ Became lower after my final menstrual period<br>○ Became higher after my final menstrual period<br>○ Other
4.4.1	***If 'Compared to before my final menstrual period, my HbA1c:' is equal to 'Other' answer this question:***<br>If other, please specify:<br>*Field type*: Textfield<br>*Variable name*: engelsAnders_HbA1c<br>*Field required*: Required	
4.5	***If 'Which type of diabetes do you have?' is equal to 'Type 1 diabetes' answer this question:***<br>Compared to before my final menstrual period, my 'Time in Range ':<br>*Field type*: Radiobutton<br>*Variable name*: engelsTIR<br>*Field required*: Required<br>*Option group name*: higher/lower/the same	○ Did not change after my final menstrual period<br>○ Became lower after my final menstrual period<br>○ Became higher after my final menstrual period<br>○ Other
4.5.1	***If 'Compared to before my final menstrual period, my 'Time in Range ':' is equal to 'Other' answer this question:***<br>If other, please specify:<br>*Field type*: Textfield<br>*Variable name*: engelsAnders_TIR<br>*Field required*: Required	
	The following questions are about fluctuations of blood sugar levels during menopause	

4.6	***If 'Which type of diabetes do you have?' is equal to 'Type 1 diabetes' answer this question:***<br>Compared to before my final menstrual period, my blood sugar levels fluctuate: (Possible answers: 1. Much less, 2. A little less, 3. The same, 4. A little more, 5. Much more)<br>1: Much less 2. A little less 3. The same 4. A little more 5. Much more<br><br>*Field type*: Slider<br>*Variable name*: engelsglucoseschommelingen<br>*Field required*: Required<br>*Field min*: 1<br>*Field min Label*: Much less after my final menstrual period<br>*Field max*: 5<br>*Field max Label*: Much more after my final menstrual period	Much less after my final menstrual period (1) —— Much more after my final menstrual period (5)
4.7	***If 'Which type of diabetes do you have?' is equal to 'Type 1 diabetes' answer this question:***<br>Compared to before my final menstrual period, I have:<br>*Field type*: Radiobutton<br>*Variable name*: engelsuitschieters_boven<br>*Field required*: Required<br>*Option group name*: hyperglycemia	◯ More hyperglycaemic episodes (hypers) after my final menstrual period<br>◯ Less hyperglycaemic episodes after my final menstrual period<br>◯ The same amount of hyperglycaemic episodes after my final menstrual period
4.8	***If 'Which type of diabetes do you have?' is equal to 'Type 1 diabetes' answer this question:***<br>Compared to before my final menstrual period, I have:<br>*Field type*: Radiobutton<br>*Variable name*: engelsuitschieters_beneden<br>*Field required*: Required<br>*Option group name*: hypoglycaemia	◯ More hypoglycaemic episodes (hypos) after my final menstrual period<br>◯ Less hypoglycaemic episodes after my final menstrual period<br>◯ The same amount of hypoglycaemic episodes after my final menstrual period
4.9	***If 'Which type of diabetes do you have?' is equal to 'Type 1 diabetes' answer this question:***<br>Compared to before my final menstrual period, my 'Time in Range' fluctuations:<br>*Field type*: Radiobutton<br>*Variable name*: engelsTIR_schommelingen<br>*Field required*: Required<br>*Option group name*: engels_Schommelen	◯ Stayed the same after my final menstrual period<br>◯ Became more after my final menstrual period<br>◯ Became less after my final menstrual period<br>◯ Other

4.9.1	***If 'Compared to before my final menstrual period, my 'Time in Range' fluctuations:' is equal to 'Other' answer this question:***<br>If other, please specify:<br>*Field type*: Textfield<br>*Variable name*: engelsAnders_TIR_schommelingen<br>*Field required*: Required	
	The following questions are about your medication for type 1 diabetes during menopause	
4.10	***If 'Which type of diabetes do you have?' is equal to 'Type 1 diabetes' answer this question:***<br>Compared to before my final menstrual period, I needed ..... insulin after my final menstrual period: (Possible answers: 1. Much less insulin, 2. Less insulin, 3. The same amount of insulin, 4. More insulin, 5. Much more insulin)<br>1. Much less insulin<br>2. Less insulin<br>3. The same amount of insulin<br>4. More insulin<br>5. Much more insulin<br><br>*Field type*: Slider<br>*Variable name*: engelsinsulinegebruik<br>*Field required*: Required<br>*Field min*: 1<br>*Field min Label*: Much less insulin<br>*Field max*: 5<br>*Field max Label*: Much more insulin	Much less insulin (1) — Much more insulin (5)

4.11	***If 'Which type of diabetes do you have?' is equal to 'Type 1 diabetes' answer this question:***<br>Compared to before my final menstrual period, the amount of insulin fluctuates... (Possible answers: 1. Much less, 2. Less, 3. The same, 4. More, 5. Much more)<br>1. Much less<br>2. Less<br>3. The same<br>4. More<br>5. Much more<br><br>*Field type*: Slider<br>*Variable name*: engelsinsulinegebruik_schommelen<br>*Field required*: Required<br>*Field min*: 1<br>*Field min Label*: Much less after my final menstrual period<br>*Field max*: 5<br>*Field max Label*: Much more after my final menstrual period	Much less after my final menstrual period (1) — Much more after my final menstrual period (5)
4.12	***If 'Do you agree with the above statements?' is equal to 'Yes' answer this question:***<br>Did an important event in the year before or the year after your last menstrual period change your blood sugar regulation? (Choose one or more answers)<br>*Field type*: Checkbox<br>*Variable name*: engelsandererereden_bloedsuikers<br>*Field required*: Required<br>*Option group name*: engels_anderegebeurtenis	☐ Yes, an event on a physical level<br>☐ Yes, an event on a mental level<br>☐ Yes, another type of event<br>☐ I don't know<br>☐ No
4.12.1	***If 'Did an important event in the year before or the year after your last menstrual period change your blood sugar regulation? (Choose one or more answers)' is equal to 'Yes, an event on a physical level' answer this question:***<br>Yes, an event on a physical level. Please specify:<br>*Field type*: Textfield<br>*Variable name*: engelsfysiek<br>*Field required*: Required	

4.12.2 ***If 'Did an important event in the year before or the year after your last menstrual period change your blood sugar regulation? (Choose one or more answers)' is equal to 'Yes, an event on a mental level' answer this question:***
Yes, an event on a mental level. Please specify:
Field type: Textfield
Variable name: engelsmentaal
Field required: Required

4.12.3 ***If 'Did an important event in the year before or the year after your last menstrual period change your blood sugar regulation? (Choose one or more answers)' is equal to 'Yes, another type of event' answer this question:***
Yes, another type of event. Please specify:
Field type: Textfield
Variable name: engelsandererreden
Field required: Required

4.12.4 ***If 'Did an important event in the year before or the year after your last menstrual period change your blood sugar regulation? (Choose one or more answers)' is equal to 'I don't know' answer this question:***
I don't know, because:
Field type: Textfield
Variable name: engelsanders_weetikniet
Field required: Not required

4.13 ***If 'Do you agree with the above statements?' is equal to 'Yes' answer this question:***
If there are any other reasons why your blood sugar regulation may have changed during and/or after menopause, please specify:
Field type: Multiline Textfield
Variable name: engelsOpmerking_bloedsuikers
Field required: Not required

[redacted]

	Hormone replacement therapy	
5.43	***If 'Do you agree with the above statements?' is equal to 'Yes' answer this question:***<br>Did you take hormone replacement therapy around the menopause?<br>*Field type*: Radiobutton<br>*Variable name*: engelsHRT<br>*Field required*: Required<br>*Option group name*: englishYes/No	◯ Yes<br>◯ No
5.43.1	***If 'Did you take hormone replacement therapy around the menopause?' is equal to 'Yes' answer this question:***<br>Did you take hormone replacement therapy because of menopause symptoms?<br>*Field type*: Radiobutton<br>*Variable name*: engelsHRT_overgangsklachten<br>*Field required*: Required<br>*Option group name*: englishYes/No	◯ Yes<br>◯ No
5.43.2	***If 'Did you take hormone replacement therapy around the menopause?' is equal to 'Yes' answer this question:***<br>Did you take hormone replacement therapy with pills, a skin patch, a spray or a gel? (Choose one or more answers)<br>*Field type*: Checkbox<br>*Variable name*: engelsHRT_soort<br>*Field required*: Required<br>*Option group name*: pills/patch/spray/gel	☐ Pills<br>☐ Patch<br>☐ Spray<br>☐ Gel

5.43.2.1	***If 'Did you take hormone replacement therapy with pills, a skin patch, a spray or a gel? (Choose one or more answers)' is equal to 'Pills' answer this question:***<br>Do you know the name of the pills you took? (Choose one or more answers)<br>*Field type*: Checkbox<br>*Variable name*: engelsHRT_tablet<br>*Field required*: Required<br>*Option group name*: engelsestrogeen-oraal	☐ Estradiol<br>☐ Estrofem<br>☐ Progynova<br>☐ Zumenon<br>☐ Femoston (estradiol/dydrogesteron)<br>☐ Trisequens (estradiol/norethiseron)<br>☐ Activelle (estradiol/norethiseron, continuous)<br>☐ Kliogest (estradiol/norethiseron, continuous)<br>☐ I don't know<br>☐ Other
5.43.2.1.1	***If 'Do you know the name of the pills you took? (Choose one or more answers)' is equal to 'Other' answer this question:***<br>If other, please specify:<br>*Field type*: Textfield<br>*Variable name*: engelsHRT_tabletten_anders<br>*Field required*: Required	
5.43.2.2	***If 'Did you take hormone replacement therapy with pills, a skin patch, a spray or a gel? (Choose one or more answers)' is equal to 'Patch' answer this question:***<br>Do you know the name of the patch? (Choose one or more answers)<br>*Field type*: Checkbox<br>*Variable name*: engelsHRT_pleister<br>*Field required*: Required<br>*Option group name*: engelspleisters-naam	☐ Estradiol patch<br>☐ Systen patch<br>☐ I don't know<br>☐ Other
5.43.2.2.1	***If 'Do you know the name of the patch? (Choose one or more answers)' is equal to 'Other' answer this question:***<br>If other, please specify:<br>*Field type*: Textfield<br>*Variable name*: engelsanders_pleister<br>*Field required*: Required	
5.43.2.3	***If 'Did you take hormone replacement therapy with pills, a skin patch, a spray or a gel? (Choose one or more answers)' is equal to 'Spray' answer this question:***<br>Do you know the name of the spray? (Choose one or more answers)<br>*Field type*: Checkbox<br>*Variable name*: engelsHRT_spray<br>*Field required*: Required<br>*Option group name*: Engels-spray-oestrogeen	☐ Lenzetto<br>☐ Other<br>☐ I don't know
5.43.2.3.1	***If 'Do you know the name of the spray? (Choose one or more answers)' is equal to 'Other' answer this question:***<br>If other, please specify:<br>*Field type*: Textfield<br>*Variable name*: engelsanders_spray<br>*Field required*: Required	

5.43.2.4	***If 'Did you take hormone replacement therapy with pills, a skin patch, a spray or a gel? (Choose one or more answers)' is equal to 'Gel' answer this question:***<br>Do you know the name of the gel? (Choose one or more answers)<br>*Field type*: Checkbox<br>*Variable name*: engelsHRT_gel<br>*Field required*: Required<br>*Option group name*: Engels-gel-oestrogeen	☐ Oestrogel<br>☐ Other<br>☐ I don't know
5.43.2.4.1	***If 'Do you know the name of the gel? (Choose one or more answers)' is equal to 'Other' answer this question:***<br>If other, please specify:<br>*Field type*: Textfield<br>*Variable name*: engelsanders_gel<br>*Field required*: Required	

Blood sugar regulation during menopause - Sleep questionnaire

Number	Question	Answers
6.1	***If 'Do you agree with the above statements?' is equal to 'Yes' answer this question:***<br>Munich Chronotype Questionnaire (MCTQ) instructions:<br>*Field type*: Summary<br>*Variable name*: instructions_1<br>*Field required*: Not required	
6.2	***If 'Do you agree with the above statements?' is equal to 'Yes' answer this question:***<br>In the last 3 months, I worked as a shift worker.<br>*Field type*: Radiobutton<br>*Variable name*: shiftworker_1<br>*Field required*: Required<br>*Option group name*: Yes-No	◯ Yes<br>◯ No
6.3	***If 'Do you agree with the above statements?' is equal to 'Yes' answer this question:***<br>I work ... days a week. (This includes unpaid work, for example, being a housewife or househusband.)<br>*Field type*: Numeric field<br>*Variable name*: work_days_1<br>*Field required*: Required<br>*Field min*: 0<br>*Field max*: 7<br>*Measurement Unit*: days	______ days
	Please consider if your sleep times may nonetheless differ between regular ‘workdays’ and ‘weekend days’ and fill out the MCTQ in this respect.	
	Workdays	
6.4	***If 'Do you agree with the above statements?' is equal to 'Yes' answer this question:***<br>On work days (or days with set appointments) I fall asleep at...<br>This is NOT the time you go to bed, but the time you actually fall asleep.<br><br>*Field type*: Time<br>*Variable name*: sleeptime_2<br>*Field required*: Required	____:____ *(hh:mm)*

6.5	***If 'Do you agree with the above statements?' is equal to 'Yes' answer this question:***<br>On work days (or days with set appointments) I wake up at...<br>This is NOT the time you get out of bed, but the time you actually wake up<br><br>*Field type*: Time<br>*Variable name*: wakeup_2<br>*Field required*: Required	[ ]:[ ] *(hh:mm)*
	Free days	
6.6	***If 'Do you agree with the above statements?' is equal to 'Yes' answer this question:***<br>On free days I actually get ready to fall asleep at...<br>This is NOT the time you go to bed, but the time you actually fall asleep.<br><br>*Field type*: Time<br>*Variable name*: sleeptime_1_1<br>*Field required*: Required	[ ]:[ ] *(hh:mm)*
6.7	***If 'Do you agree with the above statements?' is equal to 'Yes' answer this question:***<br>On free days I wake up at...<br>This is NOT the time you get out of bed, but the time you actually wake up<br><br>*Field type*: Time<br>*Variable name*: wakeup_1_1<br>*Field required*: Required	[ ]:[ ] *(hh:mm)*
